# Understanding the Latent Structure of Dynamic Risk: Seeking Empirical Constraints on Theory Development Using the VRS-SO and the Theory of Dynamic Risk

**DOI:** 10.1177/10790632211002858

**Published:** 2021-04-05

**Authors:** Mark E. Olver, David Thornton, Sarah M. Beggs Christofferson

**Affiliations:** 1 University of Saskatchewan, Saskatoon, Canada; 2 Forensic Assessment, Training, & Research (FAsTR), Madison, WI, USA; 3 University of Canterbury, Christchurch, New Zealand

**Keywords:** theory of dynamic risk, sexual recidivism, VRS-SO, risk assessment

## Abstract

The present study is part of a larger project aiming to more closely integrate theory with empirical research into dynamic risk. It seeks to generate empirical findings with the dynamic risk factors contained in the Violence Risk Scale—Sexual Offense version (VRS-SO) that might constrain and guide the further development of Thornton’s theoretical model of dynamic risk. Two key issues for theory development are (a) whether the structure of pretreatment dynamic risk factors is the same as the structure of the change in the dynamic risk factors that occurs during treatment, and (b) whether theoretical analysis should focus on individual dynamic items or on the broader factors that run through them. Factor analyses and item-level prediction analyses were conducted on VRS-SO pretreatment, posttreatment, and change ratings obtained from a large combined sample of men (*N*s = 1,289–1,431) convicted and treated for sexual offenses. Results indicated that the latent structure of pretreatment dynamic risk was best described by a three-factor model while the latent structure of change items was two dimensional. Prediction analyses examined the degree to which items were predictive beyond prediction obtained from the broader factor that they loaded on. Results showed that for some items, their prediction appeared to be largely carried by the three broad factors. In contrast, other items seem to operate as funnels through which the broader factors’ predictiveness flowed. Implications for theory development implied by these results are identified.

Over the last two decades, research into sexual recidivism risk has progressed in important ways. Achievements include identifying both static risk indicators (e.g., Hanson & Morton-Bourgon, 2005) and meaningful psychological risk factors (Mann et al., 2010) and combining these into scales designed to assess risk for sexual recidivism (e.g., Hanson et al., 2010; Hanson & Thornton, 2000; Helmus et al., 2012; Thornton et al., 2003). Furthermore, measurements of change in dynamic risk factors have also been shown to have incremental predictive value (Olver et al., 2007; Olver, Mundt, et al., 2018). To date, a key limitation of this research program has been its atheoretical nature. Ward and Beech (2015) have gone so far as to describe the construct of “dynamic risk factors” as a “theoretical dead end” while Thornton (2016) has described current understandings of dynamic risk factors as theoretically shallow.

Ward and colleagues’ critique emphasizes that current ways of understanding dynamic risk factors combine different kinds of theoretical constructs in an incoherent and clinically unhelpful way. Their response to this situation has been to develop a theoretical framework which draws heavily on the framework for investigation articulated in the Research Domain Criteria project (Cuthbert & Kozak, 2013; Ward & Fortune, 2016). Their Dynamic Risk Research Framework (DRRF) distinguishes four levels of analysis (biological, behavioral, phenomenological, and contextual) as well as six systems within which causal processes may be located (negative affective systems, positive affective systems, cognitive systems, intrapersonal social processes, self-regulation, and interpersonal social systems).

An obstacle to the DRRF approach is that adopting it seems to imply abandoning the research program on which current assessment technology is based and inventing new methodologies that allow measurement of the variables the framework regards as significant. From a DRRF perspective, no current instrument assessing dynamic risk measures theoretically meaningful constructs. Rather, they are composite constructs that refer to a grab bag of disparate causal mechanisms, contextual features, and state variables, and consequently no research with these measures is going to have theoretical meaning. Instead, they need to be deconstructed into multiple underlying causal processes with data collected at the different levels identified by the framework. Using the DRRF in conjunction with the current ways of measuring dynamic risk would be like trying to use a theoretical model of the molecular structure of materials to explain the flight of an eagle.

The DRRF also throws the clinical meaning of current scales into question. Proponents of current measures of dynamic risk see them as indicators of treatment and supervision targets (Brankley et al., 2021). However, from a DRRF perspective, a dynamic risk factor may arise from any of a disparate array of underlying causal systems and so identifying the presence of particular dynamic risk factors is not sufficient to identify appropriate targets for intervention.

It is possible that the DRRF approach may in the future develop an empirically successful research program and that using it as a clinical framework may eventually lead to demonstrably more helpful forms of clinical work. However, the DRRF is so rich in possible sources of explanation and contains so few constraints that a clinician or evaluator adopting this approach would be liable to fall into something that resembles unstructured clinical judgment. This is a significant concern since unstructured clinical judgment has been found to be the least effective form of assessment (Hanson & Morton-Bourgon, 2009). Thus, while the DRRF may offer promise for the future, at the present time, it seems just as likely to impair clinical practice.

Our preferred approach is to seek to develop ways of theoretically understanding existing measures of dynamic risk that allow them to be built on rather than abandoned. We do not think that available theory is sufficiently developed to allow clearly specified, falsifiable, hypotheses to be formulated and tested with current empirical tools. Nevertheless, we aspire to bring theory and empirical methodologies closer together, making it easier for them to interact in fruitful ways. Specifically, we hope to achieve two things. First, we want to formulate theory in a way that allows it to be shaped by empirical research with measures of dynamic risk. Second, we want to use theory to guide research studies with existing dynamic measures so that they will be more theoretically informative. This approach is different in kind from traditional empirical studies using these measures since the latter focus on evaluating the predictive value of particular measures without regard to how theoretically informative the results are.

For this project, we have chosen to use Thornton’s Theory of Dynamic Risk (TDR: Thornton, 2016; Thornton et al., 2017) as the theoretical approach to be developed and to combine this with Olver et al.’s (2007) Violence Risk Scale—Sexual Offense (VRS-SO) version. Thornton’s TDR has the advantage that dynamic risk factors can more easily be incorporated in it, relative to alternative theoretical approaches such as the Motivation-Facilitation model (Seto, 2019). The VRS-SO has the advantage that its distinction between initial dynamic risk and change is theoretically meaningful within the TDR.

Thornton (2016) provides a full account of the TDR and its three key elements of human agency, schema, and change. Under the heading of human agency, purposive behavior is understood distally through primary human goods (Ward & Gannon, 2006; Ward & Stewart, 2003) and proximally through a slightly modified application of the theory of reasoned action (Fishbein & Ajzen, 2010) so that the attractiveness of an action is understood to result from expected outcomes, social pressure, and self-efficacy. Schema are understood as developing when particular primary human goods are sought repeatedly in a given context. Following Beck’s (1996) concept of a schema mode, schema are regarded as involving triggering components (which scan for contextual elements indicating that the schema is relevant), a model of features of the environment that impede or facilitate the goals the schema is oriented around, motivational urges, and related behavioral scripts and strategies. Long-term vulnerabilities (LTVs) relevant to sexual offending arise when the activation of dominant schema leads to sexual offending, or behaviors that lead toward sexual offending, becoming more attractive. Schema regulation is required when conflicting schema are simultaneously activated or when contingencies change so that what was functional at one time is no longer functional. Treatment can be understood as augmenting natural schema regulation processes when these have been insufficient. Change may also occur as the person repeatedly pursues prosocial or antisocial ways of dealing with their current environment. This leads to the gradual development of resources supportive of the kinds of action that are repeated. Within the TDR, resources are understood to include building up schema that support and automate actions, developing schema that automate ways of dealing with negative affect caused by the course of action, and developing a social network that is supportive of these actions.

The VRS-SO is a structured rating instrument with three scales: static risk, pretreatment dynamic risk, and a change scale. Items in the change scale use a modified version of the Stages of Change model (SoC; Prochaska et al., 1992) to describe the degree to which those dynamic risk factors identified as present in the pretreatment ratings are now being regulated. In terms of the TDR, the initial dynamic risk scale is largely measuring LTVs that are embedded in the individual’s schema while items in the static scale can be understood as markers for the historical expression of individuals’ LTVs (Ward & Beech, 2004). One of the ways the VRS-SO and the TDR fit well together is that both see change occurring during treatment as involving something qualitatively distinct from the LTVs that are captured by the pretreatment assessment of dynamic risk. This contrasts with instruments like the STABLE-2007 (Fernandez et al., 2014) or the SOTIPS (McGrath et al., 2012) where change is understood and measured as an alteration in the same dynamic risk variables as would be assessed prior to treatment.

Within the project of creating a fruitful interaction between the VRS-SO and the TDR, a particular kind of preliminary research becomes desirable. The concern is not with improved prediction of recidivism. Nor is it with testing specific hypotheses derived from the TDR. Rather, the intent is to develop empirical findings that may clarify what the proper targets for theoretical explanation are and by doing so to provide constraints on how theory development using the TDR should proceed. This is the focus of this article. In particular, our analyses seek to investigate first whether individual items or broader factors should be the targets of theoretical explanation and second to clarify the nature of the factors that run through three kinds of predictive items (static, pretreatment dynamic, and change) and the relationship between these factors.

Neither the VRS-SO itself nor the TDR provide guidance on whether items or broader factors should be the level at which theoretical explanation is developed. The VRS-SO consists of items that are combined into scales. Should explanatory attention be focused at the level of these items or at a broader level? In developing the TDR Thornton (2016) described how the theory can make sense of individual psychological risk factors identified in Mann et al.’s (2010) meta-analysis. This is closer to the level of items but there is no reason why the theory could not be applied to broader factors.

Factor analysis of static actuarial risk instruments has yielded fairly consistent results. Factors representing general criminality and sexual criminality, along with a third factor that may represent the effect of age or may represent a different form of sexual deviance, have been identified (Allen & Pflugradt, 2014; Brouillette-Alarie et al., 2016; Olver et al., 2016; Roberts et al., 2002). Perhaps, it is these broader factors that should be the focus of explanation?

Further supporting the relevance of broad factors as opposed to individual items is the fact that somewhat similar factors have been in found in analyses of the items in dynamic risk instruments. Olver et al. (2007) described the VRS-SO dynamic risk items as loading onto three factors: sexual deviance, criminality, and treatment responsivity. Brouillette-Alarie and Hanson (2015) found that STABLE-2007 items load on to a general propensity for rule violation (criminality) factor and a sex crime-specific problems (sexual deviance) factor. The failure of the treatment responsivity factor to appear in STABLE-2007 factor analyses reflects that in the VRS-SO, this factor is loaded heavily on by items reflective of cognitive distortions versus insight. Items of this kind were dropped from the STABLE-2007 during the scale development process even though, paradoxically, subsequent studies have found the dropped items to be predictive (see Helmus et al., 2013). Later studies with the dynamic items from the VRS-SO (Beggs & Grace, 2010; Olver, Neumann, et al., 2018) have generated factor solutions that are broadly similar to Olver et al.’s original results, with the three substantive factors emerging, but differ in some particulars; for instance, Beggs and Grace (2010) added a three-item fourth factor termed “self-management” to marginally improve model fit (from RMSEA = .091–.072). The mere existence of broader factors does not, however, mean that items lack theoretical interest. Helmus and Thornton (2015) showed that each Static-99R item has predictive value over and above all the other items. This incremental predictive value suggests that risk might run through the individual items rather than solely through broader factors.

## Current Study and Rationale

This article uses factor analyses, together with univariate and multivariate Cox regression predicting sexual recidivism, to accumulate evidence speaking to whether theoretical analyses of risk should focus on items, on broader factors, or needs to attend to both. Evidence encouraging theoretical attention to individual items would include their having significant univariate predictive value and the evidence would be stronger if they also showed incremental predictive value relative to other items. If they showed univariate but not incremental predictive value, this would suggest that risk was actually carried by variance that they shared with other items and so that theoretical attention should be directed to that shared variance.

The factor analyses are intended to identify, and allow the description of, potentially important variance shared between items. Factors that underlie dynamic risk items would offer one kind of theoretical interest if they predicted sexual recidivism and a different kind of interest if they captured aspects of items that were unrelated to recidivism. A factor would be a particularly strong focus of theoretical interest if the factor had predictive value, but its component items showed little incremental prediction relative to the factor. In this case, it would be the shared variance described by the factor that had to be theoretically attended to in understanding dynamic risk, not the specific items.

Turning to this article’s other focus, with regard to factor analysis of pretreatment dynamic items, we hope to resolve some of the variation in findings between different samples by combining them and adding a further sample (Sowden & Olver, 2017) so that the resulting *N* is substantially larger. Beyond this, particular interest attaches to the similarity of the factors found in different kinds of items. Are the factors found in static risk items, in pretreatment dynamic risk items, and in change items, all the same? The TDR suggests that factors in static items and in pretreatment dynamic items should at least be conceptually similar since they are understood as reflecting the same LTVs, albeit through different temporal and methodological lenses. Prior research supports this but interpreting static and dynamic items as expressions of the same LTVs also implies either that conceptually related static and dynamic items will load on the same factor or, that they will load on oblique factors that correlate substantially with each other.

The TDR also suggests that the factor structure of change items might differ from the factor structure of static or pretreatment dynamic items as the former should reflect, not just the LTVs, but the structure of the environmental processes generating feedback. Such a finding would suggest that change needs to be understood separately from the ways LTVs are understood. In contrast, if similar factors are found in all three kinds of item, it would suggest that a single set of explanations would suffice.

## Method

### Samples

The present study featured a combined sample of 1,431 men incarcerated for sexual offenses who participated in sexual offense-specific treatment, of whom 1,289 had complete risk, change, and outcome data. The sample is an amalgamation of four correctional samples: three from Correctional Service of Canada (CSC) and one from New Zealand Department of Corrections. Two of these samples featured consecutive admissions to a high-intensity sexual violence reduction program, the Clearwater Sex Offender Program, with one cohort from 1983–1997 (Olver et al., 2007) and the other from 1997–2001 (Sowden & Olver, 2017). The third Canadian sample was a multisite sample of 712 men who participated in services through CSC’s National Sex Offender Program (NaSOP) low-, moderate-, or high-intensity streams from 2000–2008, of whom 570 had complete risk, change, and outcome data (Olver et al., 2014; Olver, Nicholaichuk, et al., 2020). The fourth sample consisted of 218 men who attended sexual offense-specific programming from 1993–2001 through the Kia Marama Special Treatment Unit at Rolleston Prison, New Zealand (Beggs & Grace, 2010, 2011). The common thread is that these were all incarcerated adult male correctional samples who received sexual offense-specific treatment, rated pre- and posttreatment on the VRS-SO, and followed up in the community. Although the samples varied in terms of their overall risk level, treatment intensity, and proportion of individuals with child versus adult victims, results of logistic regression modeling over fixed 5- and 10-year follow-ups demonstrated that observed differences in base rates of sexual recidivism were largely accounted for by individual differences in static and dynamic risk factors and change (Olver, Mundt, et al., 2018).

The present work is part of an ongoing program of dynamic sexual violence risk assessment research featuring the predictive properties of VRS-SO risk and change scores. Ethical approval was received by the University Research Ethics Board (Beh #15-308) to link the datasets and perform secondary analyses to address the substantive research questions. Operational approval was provided by CSC and New Zealand Department of Corrections to conduct the respective individual studies combined into the current sample. Per Simmons et al.’s (2012) 21-word solution, “We report how we determined our sample size, all data exclusions (if any), all manipulations, and all measures in the study” (para. 6).

### Measures

#### Violence Risk Scale—Sexual Offense version

The VRS-SO is an empirical actuarial sexual offense risk assessment and treatment planning measure. With 7 static and 17 dynamic items, each item is rated on a 4-point (0, 1, 2, 3) ordinal scale; higher item scores represent increased risk for sexual offending. Dynamic item ratings of 2 or 3 are considered criminogenic needs to be targeted for treatment, while those with a 0 or 1 rating are considered low risk items. As noted previously, factor analyses of the VRS-SO dynamic items have demonstrated that they can be arranged into three oblique factors—Sexual Deviance, Criminality, and Treatment Responsivity—which can be used to inform case formulation and treatment planning (Olver et al., 2007). The dynamic items are structured to assess change from treatment or other credible change agents from a modified application of the SoC model (Prochaska et al., 1992). Five SoC models have been operationalized for each dynamic item: precontemplation, contemplation, preparation, action, and maintenance. Movement from one stage to the next, in the direction of progress, is given a 0.5-point deduction, two stages of movement, a 1-point deduction and so on; an exception is movement from precontemplation to contemplation (no point allocation) given that there is no behavioral change. SoC ratings are assigned to items with 2- or 3-point ratings; a change score can then be computed through summing the SoC ratings.

In the present sample, strong interrater reliability was obtained for VRS-SO dynamic scores on randomly selected double-coded cases (intraclass correlation coefficient, single measure, consistency agreement, ICC_C1_), which were reported as follows: Olver et al. (2007) ICC_C1_ = .74 (pre), .79 (post), .68 (change); Sowden and Olver (2017) ICC_C1_ = .86 (pre, .73 with outlier), .87 (post, .74 with outlier), .84 (change, .83 with outlier); Beggs and Grace (2010) ICC_C1_ = .90 (pre), .92 (post). No ICC was available for Olver et al. (2014; Olver, Nicholaichuk, et al., 2020) since these were VRS-SO field ratings.

#### Static-99R

Static-99R (Hanson & Thornton, 1999, 2000; Helmus et al., 2012) is a 10-item static actuarial sexual violence risk assessment tool and the most frequently used measure of its nature (Bourgon et al., 2018; Kelley et al., 2020). Possible scores range from −3 to 12 and item content includes sexual and nonsexual offense history and perpetrator and victim demographics. Results from meta-analysis support the predictive accuracy of Static-99R for sexual recidivism (AUC = .72; Helmus et al., 2012). In the present sample, strong interrater reliability was obtained for Static-99R scores on randomly selected double-coded cases (ICC_C1_) which were reported as follows: Olver et al. (2007) ICC_C1_ = .84; Sowden and Olver (2017) ICC_C1_ = .97. No ICC was available for Olver et al. (2014; Olver, Nicholaichuk, et al., 2020) since these were Static-99R field ratings, or for Beggs and Grace (2010, 2011) as reliability was only examined for dynamic items.

#### Recidivism

Sexual recidivism was operationalized as any criminal charge or conviction for a new sexually motivated offense, whether this was contact or non-contact. Three samples used criminal convictions to define sexual recidivism while the NaSOP sample definition included criminal charges. Offenses that were adjudicated as nonsexual crimes that could later be determined to be sexually motivated were coded as sexual offenses; typically, this could only be determined for men who returned to federal custody in the Canadian samples for whom any new offense-related information would be documented on file (e.g., Criminal Profile Report). Recidivism criteria were coded in a binary (1-0, yes-no), while the date of new charge or conviction was coded to calculate follow-up time to permit survival analysis. Periods of time for pretrial custody were subtracted off the survival time to generate a more accurate estimate of time free in the community; time served for nonsexual offenses was not used as this information had not been consistently coded across samples (see Note 2 in section “Planned Analyses”).

### Procedure

The VRS-SO was rated pretreatment and posttreatment blind to outcome by trained raters. For three samples (the two Clearwater samples and Kia Marama), these were archival retrospective ratings from comprehensive institutional file information. The NaSOP data were prospective VRS-SO field ratings completed by program delivery staff; these ratings were extracted from a combination of treatment reports, hard copy score sheets, and Excel files. Recidivism data were obtained from official criminal records and coded independently of the VRS-SO protocols. For the Canadian samples, these came from the Canadian Police Information Centre (CPIC), Canada’s official criminal record database maintained by the Royal Canadian Mounted Police. Outcome data for the New Zealand sample came from their national register.

### Planned Analyses

The analyses proceeded in a series of phases to elucidate the structure of dynamic sexual violence risk as measured by LTVs, and to identify key risk markers that could be of particular salience both clinically and theoretically, through examining their associations (including changes therein) with sexual recidivism. First, we conducted an updated factor analysis of the VRS-SO static and dynamic items through two sets of exploratory factor analyses (EFAs) on the total combined sample of men who participated in sexual offense treatment from the four nonoverlapping samples, described previously, using Mplus version 7.4 (Muthén & Muthén 2013). The first EFA included both static and dynamic items, rated pretreatment—complete static and dynamic item data were available for the three Canadian samples (*N* = 1,183).^1^ A second EFA followed that was limited to the pretreatment dynamic items for which individual item data were available for all four samples (*N* = 1,431). Although factor structures have been established on the VRS-SO dynamic items through prior research (Olver et al., 2007; Olver, Neumann, et al., 2018), exploratory, as opposed to confirmatory, procedures were used here to identify the best fitting model generated from the data, in contrast to specifying a given model a priori and then adjusting the model parameters in a post hoc fashion to maximize fit. This approach was taken given that (a) the study featured a large combined sample, with a new sample added (Sowden & Olver, 2017), which could impact variable intercorrelations and the composition of resulting latent dimensions; and (b) given that some of the planned factor analyses (e.g., static and dynamic) had not yet been conducted on the VRS-SO items (i.e., so there was no a priori model to test).

As these factor analyses were conducted for categorical variables, when this is specified in the model, the default procedures for Mplus are to generate a polychoric correlation matrix with robust weighted least squares model estimation and oblique Geomin rotation to extract and rotate the factors to obtain a final solution. The tenability of the factor solution was evaluated according to three sets of criteria to balance fit and parsimony. First, model fit was evaluated via the comparative fit index (CFI) and the root mean squared error of approximation (RMSEA). We followed the guidelines of Marsh et al. (2010) who note that CFI values of .90 and .95 represent “acceptable” and “excellent” fit, respectively, and RMSEA values below .05 and .08 represent “close” and “reasonable” fit, respectively. Second, a factor loading cutoff of .320, amounting to 10% of variance accounted for by an item loading on a given factor, was employed (Tabachnick & Fidell, 2007). Finally, parallel analysis was conducted in Mplus to examine the potential for under extraction or over extraction of factors. Parallel analysis generates random eigenvalues for the data rank ordered by magnitude, which in turn are evaluated against the rank ordered eigenvalues for factors extracted from the data. The point at which the eigenvalue magnitude for a randomly generated factor exceeds that for the similar ranked actual factor indicates maximum number of allowable factors to be retained.

Second, to better understand the nature of the LTVs that underpin VRS-SO scores, finer grained predictive accuracy analyses were executed to examine the prediction of sexual recidivism by the individual dynamic items. All outcome analyses were conducted on the combined sample of 1,289 cases with complete pre-post and recidivism data. We used Cox regression survival analysis for the bulk of our prediction analyses in order to permit the use of the entire sample and hence, recidivists, since the technique adjusts and controls for individual differences in follow-up time. Cox regression generates a hazard ratio (HR), representing the proportionate increase in the hazard of an outcome, such as recidivism, for every one-unit increase in the predictor. HR values above 1.0 indicate a positive association between the predictor and the criterion, while values below 1.0 represent an inverse association. For these analyses, we examined the prediction of sexual recidivism via a series of univariate Cox regressions at the individual item level for pretreatment, posttreatment, and change ratings. This was followed by an incremental examination of the dynamic items, pretreatment, posttreatment, and change, through simultaneous entry of all predictors, to ascertain which variables uniquely predicted this outcome.^2^

The final set of analyses focused on the examination of dynamic item change scores. We conducted an EFA of dynamic item change scores to elucidate the structure of treatment change in this sample (*N* =1,372). The same three criteria (i.e., CFI/RMSEA indices, loading magnitude, parallel analysis) utilized to evaluate the factor structure for the static and dynamic and dynamic only models were employed to evaluate the fit of the change score factor solution. Cox regression survival analysis was again employed to examine the association between change scores and possible reductions in recidivism, with the expectation being HRs < 1.0. Given that the pretreatment score constrains the amount of movement that an individual can make in terms of changing on a given measure, and given the scoring rules of the VRS-SO where non-criminogenic items are not given SoC ratings (thus the change is most typically “0” for low scoring items), we employed residualized change scores, controlling for pretreatment score for all change analyses (Beggs & Grace, 2011). The change prediction analyses featured the examination of changes made on individual items to possible decreases in sexual recidivism, controlling for pretreatment score through univariate and multivariate Cox regressions, as well as the change factors derived from EFA controlling for their respective pretreatment scores. We also controlled for Static-99R as a well-established static risk measure, to provide a more stringent test of the incremental predictive validity (i.e., accounting for offense history and age), of the change factors to sexual recidivism.^3^ These analyses would inform whether the treatment changes registered on individual dynamic items cluster together, the results of which have implications for therapeutic change processes for risk reduction. We conclude with a comparison of change factor scores among men with different victim profiles (i.e., any adult victim vs. exclusively child victims) and as a function of sample and setting through computing standardized mean difference (Cohen’s *d*).

## Results

### Updated Factor Analysis of Static and Dynamic Items

From the three Canadian samples, a five-factor solution emerged (CFI = .948, RMSEA = .065, eigenvalues = 6.055, 3.926, 2.057, 1.463, 1.373). The results of parallel analysis suggested the possible presence of a sixth factor (eigenvalue from actual data 1.136), given that it was slightly higher than the average eigenvalue for the sixth factor generated from parallel analysis (1.128); however, scrutiny of the sixth factor suggested that it was a “pseudo-factor,” with only one item loading highest (D11 released to high-risk situations) and also cross loading on the General Criminality factor. As such, to balance fit and parsimony, a five-factor solution was retained. The static and dynamic items largely loaded on separate sets of factors, two static and three dynamic (see Table 1): Age, Sexual Criminality, General Criminality, Sexual Deviance, and Treatment Responsivity. The results had several parallels to previous VRS-SO factor analyses, with age and offense-related variables loading on separate factors (Olver et al., 2016), and the item composition of the dynamically based factors being isomorphic to the inaugural VRS-SO factor analysis in Olver et al. (2007). There were some exceptions as S7 prior sentencing dates loaded on the largely dynamic general criminality factor and D11 released to high-risk situations now loaded most highly on the general criminality dimension (previously loading on Treatment Responsivity). In addition, D7 emotional control and D17 intimacy deficits loaded significantly and most highly on Sexual Deviance, although they did not exceed the loading threshold for inclusion on a factor. (Both were non-loading in Olver et al.’s 2007 analysis. Of note, D17 loaded on Sexual Deviance in Beggs & Grace, 2010.) The five factors were intercorrelated at magnitudes that ranged from negligible to moderate (see Supplemental Table S1).

**Table 1.** Factor Loading Matrix for Violence Risk Scale—Sexual Offense Static and Dynamic Items.

Item	Age	Sexual criminality	General criminality	Sexual deviance	Treatment responsivity
S1 Age at release	.952*	−.006	−.005	−.119	−.022
S2 Age at first adjudicated sex offense	.667*	.285*	.221*	.039*	−.067*
S3 Sexual offense victim profile	.184*	.709*	.043	−.049	.033
S4 Prior sex offenses	−.135*	.562*	.230*	.237*	−.029
S5 Unrelated victims	.239*	.909*	−.001	−.004	.009
S6 Gender and number of victims	−.007	.683*	−.136*	.328*	.051*
S7 Prior sentencing dates	−.245*	.128*	.773*	−.182*	−.038*
D1 Sexually deviant lifestyle	.030	.079*	−.041	.832*	.013
D2 Sexual compulsivity	.015	−.089*	.132*	.767*	−.081*
D3 Offense planning	−.165*	.019	−.187*	.655*	.068*
D4 Criminal personality	.013	−.029	.570*	.157*	.170*
D5 Cognitive distortions	−.202*	−.039	.002	.030	.594*
D6 Interpersonal aggression	.049	−.101*	.799*	.003	.051
D7 Emotional control	.017	.020	.247*	.292*	−.197*
D8 Insight	−.041*	.012	.011	−.238*	.833*
D9 Substance abuse	−.050	.029	.561*	−.287*	−.126*
D10 Community support	−.011	−.046	.491*	.191*	.216*
D11 Released to high risk situations	.021	.022	.456*	.288*	.250*
D12 Sexual offending cycle	−.204*	.265*	.066*	.608*	−.036
D13 Impulsivity	.131	−.098*	.568*	−.088*	.121*
D14 Compliance with community supervision	.050	.075*	.676*	.005	.203*
D15 Treatment compliance	.077*	.061	.355*	.129*	.436*
D16 Deviant sexual preference	−.010	.144*	−.041	.764*	.054
D17 Intimacy deficits	.201*	−.027	.098	.261*	−.058

*Note. N* = 1,183. Items loading significantly marked with an asterisk. Items denoted as loading on a given factor in bold italics. Highest loading for an item with respect to a given factor in italics.

The EFA was repeated a second time, this time being limited to the dynamic items, for which individual item data were available for all four samples. Three latent dimensions emerged as previously (CFI = .934, RMSEA = .077, eigenvalues = 4.369, 3.458, 1.668) and with high consistency to Olver et al. (2007) and Olver, Neumann, et al. (2018). The results of parallel analysis suggested the possible presence of a fourth factor (eigenvalue from actual data 1.198), given that it was slightly higher than the average eigenvalue for the fourth factor generated from parallel analysis (1.106); however, scrutiny of the fourth factor suggested that it was also a probable “pseudo-factor,” with only one item loading uniquely (again, D11 released to high-risk situations) and three other items cross loading, two of which loaded higher on another factor. As such, to balance fit and parsimony, a three-factor solution was retained. As seen in Table 2, on the Sexual Deviance dimension, D7 emotional control and D17 intimacy deficits loadings met the threshold for inclusion on the factor, General Criminality retained D11 released to high-risk situations, while Treatment Responsivity appeared to be reduced to a Cognition dimension as D15 treatment compliance cross loaded on this and General Criminality, but higher on the latter.

**Table 2.** Factor Loading Matrix for Violence Risk Scale—Sexual Offense Dynamic Items.

Item	Sexual deviance	General criminality	Treatment responsivity/cognition
D1 Sexually deviant lifestyle	.855*	.020	.005
D2 Sexual compulsivity	.646*	.237*	−.046
D3 Offense planning	.698*	−.247*	.113*
D4 Criminal personality	.123*	.582*	.076
D5 Cognitive distortions	−.034	−.046	.671*
D6 Interpersonal aggression	−.009	.772*	−.145*
D7 Emotional control	.323*	.297*	−.228*
D8 Insight	−.321	.012	.842*
D9 Substance abuse	−.225	.539*	−.189*
D10 Community support	.001	.562*	.248*
D11 Released to high-risk situations	.106*	.531*	.313*
D12 Sexual offending cycle	.655*	.124*	.026
D13 Impulsivity	−.196*	.681*	.019
D14 Compliance with community supervision	−.047	.726*	.170*
D15 Treatment compliance	.056	.454*	.380*
D16 Deviant sexual preference	.834*	−.028	−.009
D17 Intimacy deficits	.352*	.024	−.119*

*Note. N* = 1,431. Items loading significantly marked with an asterisk. Items denoted as loading on a given factor in bold italics.

### LTV Associations With Sexual Recidivism

The aggregate sample was followed up an average 12.3 (*SD* = 4.4) years post release, during which the base rate of sexual recidivism was 16.9% (*n* = 218/1,289). Individual VRS-SO dynamic predictors of sexual recidivism are arranged in descending order by HR magnitude (Table 3). Almost all items, with a few exceptions, demonstrated significant predictive accuracy; only pretreatment measured D17 intimacy deficits, D16 deviant sexual preference, D5 cognitive distortions, and both pre- and posttreatment measured D3 offense planning, did not significantly predict sexual recidivism. Thus, from a pure prediction standpoint, most items contribute important information regarding the potential for future sexual violence. Change scores on each of the dynamic items, controlling for pretreatment score, were significantly associated with decreased sexual recidivism. Next, Cox regression survival analysis was conducted on the entire sample to examine the incremental prediction of sexual recidivism over time, controlling for each predictor. Given that D17 intimacy deficits item ratings were not available for the entire sample, the analyses were limited to the 16 core dynamic items. As seen in Table 4, across pre- and post-analysis, consistent unique predictors of outcome were D1 sexually deviant lifestyle, D8 insight, D9 substance abuse, and D14 compliance with community supervision; D15 treatment compliance uniquely predicted outcome in pretreatment, but not posttreatment, analyses. Similarly, residual change scores for D1 sexually deviant lifestyle and D9 substance abuse also incrementally predicted decreased sexual recidivism, as did changes on D3 offense planning and D13 impulsivity (Table 4).

**Table 3.** Univariate Cox Regression Survival Analysis: VRS-SO Dynamic Item and Change Score Associations With Sexual Recidivism.

Pretreatment item		HR	95% CI	Posttreatment item		HR	95% CI	Item change score		HR	95% CI
D14	Compliance with community supervision	1.65***	[1.46, 1.86]	D14	Compliance with community supervision	1.81***	[1.58, 2.07]	D13	Impulsivity	0.17***	[0.09, 0.32]
D15	Treatment compliance	1.58***	[1.38, 1.79]	D15	Treatment compliance	1.72***	[1.49, 1.98]	D10	Community support	0.19***	[0.11, 0.35]
D11	Released to high-risk situations	1.47***	[1.28, 1.69]	D11	Released to high-risk situations	1.63***	[1.41, 1.89]	D14	Compliance with community supervision	0.21***	[0.10, 0.41]
D6	Interpersonal aggression	1.44***	[1.27, 1.64]	D6	Interpersonal aggression	1.56***	[1.36, 1.79]	D3	Offense planning	0.22***	[0.13, 0.35]
D10	Community support	1.39***	[1.21, 1.60]	D10	Community support	1.57***	[1.35, 1.82]	D2	Sexual compulsivity	0.25***	[0.13, 0.48]
D8	Insight	1.34***	[1.13, 1.60]	D8	Insight	1.53***	[1.29, 1.82]	D1	Sexually deviant lifestyle	0.25***	[0.17, 0.37]
D4	Criminal personality	1.32***	[1.16, 1.50]	D1	Sexually deviant lifestyle	1.52***	[1.31, 1.75]	D4	Criminal personality	0.26***	[0.13, 0.53]
D13	Impulsivity	1.31***	[1.16, 1.49]	D13	Impulsivity	1.49***	[1.29, 1.73]	D11	Released to high risk situations	0.26***	[0.14, 0.47]
D7	Emotional control	1.28**	[1.10, 1.49]	D7	Emotional control	1.48***	[1.24, 1.75]	D9	Substance abuse	0.27***	[0.16, 0.47]
D9	Substance abuse	1.26***	[1.13, 1.41]	D12	Sexual offending cycle	1.44***	[1.24, 1.67]	D17	Intimacy deficits	0.29***	[0.16, 0.53]
D1	Sexually deviant lifestyle	1.26***	[1.11, 1.43]	D4	Criminal personality	1.42***	[1.23, 1.65]	D12	Sexual offending cycle	0.30***	[0.19, 0.47]
D12	Sexual offending cycle	1.24***	[1.09, 1.41]	D9	Substance abuse	1.37***	[1.21, 1.56]	D5	Cognitive distortions	0.34***	[0.21, 0.53]
D2	Sexual compulsivity	1.18**	[1.05, 1.34]	D2	Sexual compulsivity	1.32***	[1.15, 1.52]	D7	Emotional control	0.34***	[0.21, 0.55]
D16	Deviant sexual preference	1.12	[1.00, 1.26]	D17	Intimacy deficits	1.23*	[1.05, 1.45]	D6	Interpersonal aggression	0.40***	[0.23, 0.67]
D17	Intimacy deficits	1.11	[0.96, 1.29]	D16	Deviant sexual preference	1.20**	[1.05, 1.36]	D16	Deviant sexual preference	0.40***	[0.25, 0.65]
D5	Cognitive distortions	1.02	[0.88, 1.19]	D5	Cognitive distortions	1.19*	[1.01, 1.41]	D8	Insight	0.45***	[0.31, 0.66]
D3	Offense planning	1.01	[0.89, 1.14]	D3	Offense planning	1.16	[1.00, 1.34]	D15	Treatment compliance	0.46***	[0.28, 0.75]

*Note.* Pretreatment *N* = 1,289; posttreatment and change *N* = 1,246 (except D17 *N* = 925 and 921, respectively). Change scores are residualized scores controlling for item pretreatment score. VRS-SO = Violence Risk Scale—Sexual Offense; HR = hazard ratio; CI = confidence interval.

* *p* < .05. **p < .01. ***p < .001.

**Table 4.** Cox Regression Survival Analyses: Incremental Predictive Validity for Sexual Recidivism by Dynamic Item Pre, Post, and Change Ratings.

Pretreatment item		*B*	*SE*	*p*	*e^B^*	95% CI	Posttreatment item		*B*	*SE*	*p*	*e^B^*	95% CI	Item change score		*B*	*SE*	*p*	*e^B^*	95% CI
**D14**	**Compliance with community sup**	**.33**	**.08**	**.001**	**1.39**	**[1.18, 1.63]**	**D1**	**Sexually deviant lifestyle**	**.41**	**.11**	**.001**	**1.50**	**[1.21, 1.86]**	**D3**	**Offense planning**	**−.89**	**.33**	**.007**	**0.41**	**[0.22, 0.85]**
**D8**	**Insight**	**.28**	**.11**	**.012**	**1.33**	**[1.06, 1.66]**	**D14**	**Compliance with community sup**	**.38**	**.09**	**.001**	**1.45**	**[1.21, 1.75]**	**D13**	**Impulsivity**	**−.84**	**.40**	**.037**	**0.43**	**[0.20, 0.95]**
**D1**	**Sexually deviant lifestyle**	**.26**	**.01**	**.008**	**1.30**	**[1.07, 1.57]**	**D8**	**Insight**	**.32**	**.12**	**.009**	**1.37**	**[1.08, 1.74]**	**D1**	**Sexually deviant lifestyle**	**−.81**	**.33**	**.015**	**0.45**	**[0.23, 0.85]**
**D15**	**Treatment compliance**	**.18**	**.09**	**.038**	**1.20**	**[1.01, 1.41]**	**D9**	**Substance abuse**	**.24**	**.08**	**.002**	**1.27**	**[1.09, 1.47]**	**D9**	**Substance abuse**	**−.72**	**.32**	**.022**	**0.49**	**[0.26, 0.90]**
**D9**	**Substance abuse**	**.16**	**.07**	**.017**	**1.18**	**[1.03, 1.34]**	D7	Emotional control	.17	.09	.077	1.18	[0.98, 1.42]	D10	Community support	−.70	.37	.072	0.51	[0.25, 1.06]
D7	Emotional control	.16	.08	.064	1.17	[0.99, 1.38]	D15	Treatment compliance	.10	.10	.336	1.11	[0.90, 1.35]	D4	Criminal personality	−.41	.41	.312	0.66	[0.30, 1.47]
D6	Interpersonal aggression	.12	.08	.132	1.13	[0.96, 1.33]	D6	Interpersonal aggression	.07	.10	.454	1.07	[0.89, 1.29]	D14	Compliance with community sup	−.34	.41	.403	0.71	[0.32, 1.58]
D12	Sexual offending cycle	.06	.08	.479	1.06	[0.90, 1.25]	D12	Sexual offending cycle	.06	.10	.507	1.07	[0.88, 1.28]	D11	Released to high-risk situations	−.34	.36	.354	0.71	[0.35, 1.46]
D11	Released to high-risk situations	.05	.10	.615	1.05	[0.87, 1.26]	D11	Released to high-risk situations	.04	.11	.737	1.04	[0.84, 1.29]	D2	Sexual compulsivity	−.16	.38	.674	0.85	[0.41, 1.79]
D16	Deviant sexual preference	.00	.08	.975	1.003	[0.85, 1.18]	D3	Offense planning	.03	.10	.764	1.03	[0.85, 1.24]	D7	Emotional control	−.07	.29	.825	0.94	[0.53, 1.66]
D3	Offense planning	−.02	.08	.811	0.98	[0.85, 1.15]	D13	Impulsivity	−.01	.10	.962	1.00	[0.82, 1.21]	D8	Insight	.09	.27	.728	1.10	[0.65, 1.87]
D2	Sexual compulsivity	−.04	.08	.675	0.97	[0.82, 1.14]	D2	Sexual compulsivity	−.05	.10	.642	0.96	[0.79, 1.16]	D16	Deviant sexual preference	.11	.30	.713	1.11	[0.63, 1.99]
D4	Criminal personality	−.04	.09	.629	0.96	[0.81, 1.14]	D10	Community support	−.05	.11	.667	0.96	[0.78, 1.18]	D12	Sexual offending cycle	.14	.33	.667	1.15	[0.61, 2.17]
D13	Impulsivity	−.05	.08	.590	0.96	[0.81, 1.13]	D4	Criminal personality	−.05	.10	.628	0.95	[0.79, 1.15]	D5	Cognitive distortions	.17	.31	.579	1.19	[0.65,2.18]
D10	Community support	−.06	.09	.529	0.94	[0.79, 1.13]	D16	Deviant sexual preference	−.07	.09	.468	0.94	[0.78, 1.12]	D6	Interpersonal aggression	.21	.33	.510	1.24	[0.66, 2.34]
D5	Cognitive distortions	−.23	.10	.016	0.79	[0.66, 0.96]	D5	Cognitive distortions	−.27	.12	.022	0.77	[0.61, 0.96]	D15	Treatment compliance	.46	.32	.149	1.58	[0.63, 1.99]

*Note.* Pretreatment *N* = 1,305, Posttreatment and Change *N* = 1,261. Items incrementally predictive highlighted in bold font. Predictors arranged in descending magnitude of *B* value. Change scores are residualized scores controlling for item pretreatment score. CI = confidence interval.

Finally, a set of Cox regressions were conducted examining the predictive associations between a given VRS-SO dynamic item (pre or post), controlling for scores on its parent factor (minus the item score). The analyses would be a further examination of the relative importance of a given item loading on its candidate factor in predicting sexual recidivism. In the majority of cases, the composite factor even with the candidate item subtracted remained predictive of outcome, while there was variability in which items continued to be uniquely predictive of outcome; however, those items that did predict were many of the same uniquely predictive items identified in Table 4. As seen in Table 5, pre- and posttreatment ratings of D1 sexually deviant lifestyle and D12 sexual offending cycle uniquely predicted sexual recidivism, controlling for Sexual Deviance factor score. Moreover, pre- and posttreatment ratings of D10 community support and D14 compliance with community supervision uniquely predicted sexual recidivism controlling for Criminality factor score, as did posttreatment D9 substance abuse ratings. Finally, pre- and posttreatment ratings of D11 released to high-risk situations and D15 treatment compliance uniquely predicted sexual recidivism controlling for Treatment Responsivity factor score, while pretreatment D5 cognitive distortions also uniquely predicted this outcome. These results were substantively unchanged after imposing an additional control for Sexual Criminality static factor score (i.e., for the Sexual Deviance item analyses) or S7 sentencing dates item (i.e., for Criminality item analyses). See Supplemental Table S2 for these additional findings. Finally, residual change scores for each item predicted decreased sexual recidivism, while also controlling for pretreatment scores on the parent factor, minus the respective pretreatment item score represented by the change association.

**Table 5.** Incremental Predictive Validity for Sexual Recidivism by Pre- and Posttreatment Rated Dynamic Items and Change Scores Controlling for Factor Score.

Item and factor		Pretreatment					Posttreatment					Change				
		*B*	*SE*	*p*	*e^B^*	95% CI	*B*	*SE*	*p*	*e^B^*	95% CI	*B*	*SE*	*p*	*e^B^*	95% CI
D1	Sexually deviant lifestyle	.27	.09	**.004**	1.31	[1.09, 1.58]	.39	.10	**<.001**	1.47	[1.20, 1.80]	−1.40	.21	**<.001**	0.25	[0.16, 0.37]
	Sexual deviance w/o D1	−.06	.03	.835	0.99	[0.94, 1.05]	.02	.03	.634	1.02	[0.95, 1.08]	0.06	.02	**.006**	1.06	[1.02, 1.10]
D2	Sexual compulsivity	.12	.08	.096	1.13	[0.98, 1.31]	.11	.09	.194	1.12	[0.94, 1.33]	−1.23	.32	**<.001**	0.29	[0.15, 0.56]
	Sexual deviance w/o D2	.04	.02	.119	1.04	[0.99, 1.09]	.09	.03	**.001**	1.09	[1.04, 1.15]	0.04	.02	**.030**	1.05	[1.004, 1.09]
D3	Offense planning	−.22	.08	**.003**	0.80	[0.69, 0.93]	−.14	.09	.112	0.87	[0.73, 1.03]	−1.35	.25	**<.001**	0.26	[0.16, 0.42]
	Sexual deviance w/o D3	.12	.02	**<.001**	1.13	[1.07, 1.18]	.15	.03	**<.001**	1.16	[1.10, 1.22]	0.06	.02	**.005**	1.06	[1.02, 1.10]
D12	Sexual offending cycle	.18	.08	**.018**	1.20	[1.03, 1.40]	.27	.09	**.002**	1.31	[1.10, 1.57]	−1.18	.23	**<.001**	0.31	[0.20, 0.48]
	Sexual deviance w/o D12	.03	.02	.295	1.03	[0.98, 1.07]	.06	.03	**.034**	1.06	[1.00, 1.11]	0.05	.02	**.008**	1.05	[1.01, 1.10]
D16	Deviant sexual preference	−.04	.08	.658	0.97	[0.83, 1.13]	−.08	.09	.350	0.92	[0.78, 1.09]	−0.85	.24	**<.001**	0.43	[0.27, 0.69]
	Sexual deviance w/o D16	.08	.03	**.006**	1.08	[1.02, 1.14]	.15	.03	**<.001**	1.16	[1.09, 1.23]	0.07	.02	**.002**	1.07	[1.02, 1.11]
D4	Criminal personality	.03	.08	.720	1.03	[0.88, 1.20]	.02	.09	.836	1.02	[0.86, 1.21]	−1.06	.32	**.001**	0.35	[0.18, 0.66]
	Criminality w/o D4	.16	.02	**<.001**	1.17	[1.12, 1.23]	.20	.03	**<.001**	1.21	[1.16, 1.28]	0.16	.02	**<.001**	1.17	[1.12, 1.22]
D6	Interpersonal aggression	.10	.08	.229	1.10	[0.94, 1.29]	.09	.09	.320	1.09	[0.92, 1.31]	−0.68	.24	**.005**	0.51	[0.32, 0.82]
	Criminality w/o D6	.15	.03	**<.001**	1.16	[1.10, 1.22]	.18	.03	**<.001**	1.20	[1.14, 1.27]	0.16	.02	**<.001**	1.17	[1.13, 1.22]
D9	Substance abuse	.09	.06	.153	1.09	[0.97, 1.23]	.16	.07	**.024**	1.17	[1.02, 1.34]	−0.94	.26	**<.001**	0.39	[0.24, 0.65]
	Criminality w/o D9	.15	.02	**<.001**	1.16	[1.11, 1.21]	.17	.02	**<.001**	1.18	[1.13, 1.23]	0.14	.02	**<.001**	1.16	[1.11, 1.20]
D10	Community support	.18	.08	**.019**	1.19	[1.03, 1.38]	.24	.08	**.004**	1.27	[1.08, 1.49]	−1.41	.30	**<.001**	0.25	[0.14, 0.44]
	Criminality w/o D10	.13	.02	**<.001**	1.14	[1.09, 1.19]	.15	.02	**<.001**	1.17	[1.12, 1.22]	0.14	.02	**<.001**	1.15	[1.11, 1.20]
D13	Impulsivity	−.01	.08	.948	1.00	[0.86, 1.16]	.04	.09	.652	1.04	[0.87, 1.24]	−1.27	.31	**<.001**	0.28	[0.15, 0.52]
	Criminality w/o D13	.17	.03	**<.001**	1.18	[1.13, 1.24]	.19	.03	**<.001**	1.21	[1.15, 1.28]	0.15	.02	**<.001**	1.16	[1.12, 1.21]
D14	Compliance with com. sup.	.38	.07	**<.001**	1.46	[1.26, 1.69]	.43	.08	**<.001**	1.53	[1.31, 1.80]	−1.30	.32	**<.001**	0.27	[0.15, 0.52]
	Criminality w/o D14	.08	.03	**.001**	1.08	[1.03, 1.14]	.11	.03	**<.001**	1.11	[1.06, 1.17]	0.14	.02	**<.001**	1.15	[1.10, 1.20]
D5	Cognitive distortions	−.18	.09	**.033**	0.83	[0.70, 0.99]	−.17	.10	.080	0.84	[0.69, 1.02]	−0.68	.24	**.005**	0.51	[0.32, 0.82]
	Txt Responsivity w/o D5	.24	.04	**<.001**	1.27	[1.19, 1.37]	.27	.04	**<.001**	1.32	[1.22, 1.42]	0.18	.03	**<.001**	1.20	[1.12, 1.29]
D8	Insight	.10	.10	.349	1.10	[0.90, 1.35]	.14	.11	.186	1.16	[0.93, 1.43]	−0.59	.20	**.004**	0.55	[0.37, 0.82]
	Txt Responsivity w/o D8	.17	.04	**<.001**	1.19	[1.10, 1.28]	.19	.04	**<.001**	1.21	[1.11, 1.31]	0.16	.03	**<.001**	1.18	[1.10, 1.26]
D11	Released to HRS	.32	.08	**<.001**	1.38	[1.19, 1.60]	.39	.09	**<.001**	1.48	[1.25, 1.75]	−1.14	.30	**<.001**	0.32	[0.18, 0.58]
	Txt Responsivity w/o D11	.09	.04	**.020**	1.09	[1.01, 1.18]	.09	.04	**.022**	1.10	[1.01, 1.18]	0.12	.04	**.001**	1.13	[1.06, 1.21]
D15	Treatment compliance	.41	.08	**<.001**	1.50	[1.29, 1.75]	.44	.09	**<.001**	1.55	[1.31, 1.85]	−0.67	.25	**.007**	0.51	[0.32, 0.83]
	Txt Responsivity w/o D15	.05	.04	.219	1.05	[0.97, 1.14]	.08	.04	**.049**	1.08	[1.00, 1.17]	0.14	.04	**<.001**	1.15	[1.07, 1.23]

*Note.* Significant *p*-values in bold font. Change scores are residualized scores controlling for item pretreatment score. CI = confidence interval.

### The Latent Structure of LTV Change

The final set of analyses examined the latent structure of VRS-SO dynamic item change scores and their associations with sexual recidivism. Table 6 reports the factor loading matrix for VRS-SO change scores for all 17 dynamic items. Good model fit was obtained (CFI = .945, RMSEA = .059) for a two-factor solution. The results of parallel analysis also supported the two-factor model; the eigenvalues for the first two factors extracted (6.414 and 2.159) were higher than the first two randomly generated average eigenvalues (1.200 and 1.162), while the eigenvalue for a third change factor extracted from the actual data (0.923) was smaller than the third randomly generated eigenvalue (1.128) from parallel analysis. The change score factors were labeled (a) Sexual Self-Management, which had item change scores from Sexual Deviance and Treatment Responsivity factors loading, and (b) Regulation of Antisociality, which resembled the original Criminality factor in form and structure. The two change factors were significantly correlated at *r* = .25 (*p* < .001). D2 sexual compulsivity change scores loaded equivalently on both factors but not at the threshold required to be retained; as such, it was not included in the computation of change scores for either factor. Univariate Cox regressions of change scores, controlling for pretreatment score (i.e., residualized change score), demonstrated that positive (i.e., prosocial) changes on each of the items were significantly associated with decreased sexual recidivism (Table 3). Cox regression survival analysis examining the incremental validity of the change factors further demonstrated that the Sexual Self-Management and Regulation of Antisociality change factors each uniquely predicted reductions in sexual recidivism, with and without controlling for Static-99R scores (Table 7).

**Table 6.** Factor Loading Matrix for Violence Risk Scale—Sexual Offense Dynamic Item Change Scores.

Item	Sexual self-management	Regulation of antisociality
D1 Sexually deviant lifestyle	.774*	.000
D2 Sexual compulsivity	.296*	.313*
D3 Offense planning	.836*	−.077
D4 Criminal personality	−.021	.517*
D5 Cognitive distortions	.447*	.420*
D6 Interpersonal aggression	.082*	.625*
D7 Emotional control	.494*	.367*
D8 Insight	.493*	.438*
D9 Substance abuse	−.010	.629*
D10 Community support	.007	.602*
D11 Released to high-risk situations	.076	.614*
D12 Sexual offending cycle	.709*	.214*
D13 Impulsivity	−.061	.653*
D14 Compliance with community supervision	−.053	.648*
D15 Treatment compliance	.072	.436*
D16 Deviant sexual preference	.583*	.074*
D17 Intimacy deficits	.686*	−.037

*Note. N* = 1,372. Items loading significantly marked with an asterisk. Items denoted as loading on a given factor in bold italics.

**Table 7.** Cox Regression Survival Analyses: Incremental Predictive Validity for Change Factor Score Domains With and Without controlling for Static-99R Score (*N* = 1,261).

Regression model		*B*	*SE*	*p*	*e^B^*	95% CI
1	Sexual self-management change	−.229	.052	<.001	.80	[0.719, 0.881]
	Regulation of antisociality change	−.170	.073	.020	.84	[0.731, 0.974]
2	Static-99R	.208	.028	<.001	1.23	[1.166, 1.301]
	Sexual self-management change	−.127	.056	.023	.88	[0.790, 0.983]
	Regulation of antisociality change	−.195	.070	.005	.82	[0.718, 0.943]

**Figure 1.**
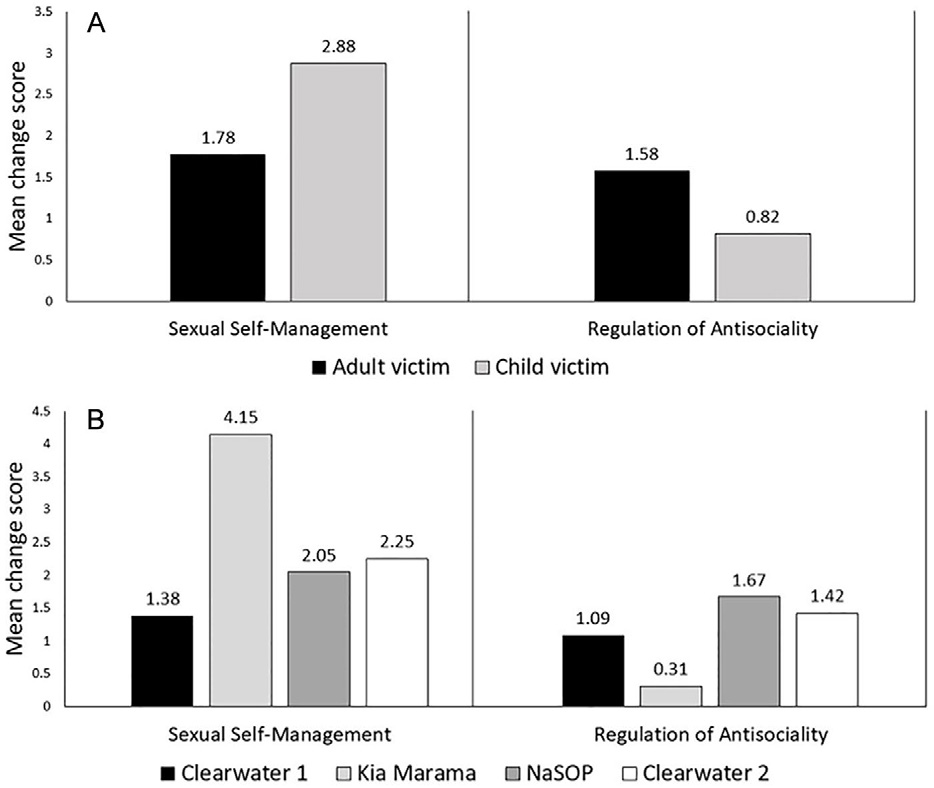
(A) Group change comparisons on Sexual Self-Management and Regulation of Antisociality domains as a function of victim profile; (B) Group change comparisons as a function of sample setting. *Note.* NaSOP = National Sex Offender Program.

Examination of change factor scores as a function of sexual offense victim profile and treatment setting were illustrative. As seen in Figure 1A, men who had adult victims scored significantly lower on the Sexual Self-Management change factor than men with exclusively child victims (*d* = −.63, *p* < .001); however, the trend was reversed for Regulation of Antisociality change factor scores, with men who had adult victims registering significantly more change in this domain (*d* = .61, *p* < .001). When group differences on the change factors were examined as a function of setting (Figure 1B), men from New Zealand’s Kia Marama Program (which is populated by men who have sexually offended against children), had significantly higher Sexual Self-Management change scores than the mixed populations from all three Canadian settings (*d*s = 1.09 to 1.97, *p* < .001), but also significantly lower Regulation of Antisociality change factor scores (*d*s = −.85 to −1.28, *p* < .001).

Finally, in supplemental analyses conducted for comparative purposes, when the original dynamic factor and change scores were examined incrementally, Sexual Deviance and Criminality pretreatment and change scores uniquely predicted sexual recidivism in the expected direction, while Treatment Responsivity pretreatment and change scores did not (see Supplemental Table S4).

## Discussion

Our factor analyses of static and initial dynamic risk items gave results consistent with earlier published work: static items loaded on to a Sexual Criminality, a General Criminality, and an Age factor while initial dynamic risk items loaded onto Sexual Deviance, General Criminality, and Treatment Responsivity factors. The one notable divergence from Olver et al.’s (2007) initial factor analytic results on which VRS-SO subscales are based is that the two more behavioral items from the Treatment Responsivity subscale (D11 release to high-risk situations and D15 treatment compliance) loaded about equally on General Criminality and Treatment Responsivity factors. Another finding not apparent from previous work was that while Sexual Criminality and Sexual Deviance appear as distinct factors, there was a single General Criminality factor loading both static and dynamic items. Importantly, although Sexual Deviance and Sexual Criminality are distinct factors, they are moderately correlated with each other (*r* = .466). It is likely that criminality would also have split into distinct static and dynamic factors if additional static items loading this factor had been included in the analysis. Olver et al. (2016) described a factor analysis that used non-redundant Static-99R and VRS-SO static items. In that study, as here, the Sexual Criminality factor correlated just under .50 with the dynamic Sexual Deviance score while a Criminality factor, defined on static items alone, correlated just over .60 with the dynamic Criminality score. Taken together, these findings are consistent with the idea that static items track the historical expression of the same LTVs that are expressed in dynamic items but can emerge as distinct but correlated factors because of different method variance. Overall, the results provide a consistent and stable picture of the structure of the LTVs emerging in static and pretreatment dynamic items measured by the VRS-SO that is consistent with the TDR.

Factor analysis of VRS-SO change ratings indicated a structure that is different from that found for LTVs. The parallel analysis decisively indicated that a two, rather than three, factor structure was appropriate. The first factor which we have labeled Sexual Self-Management was uniquely loaded by items reflecting regulation of sexual deviance while the second factor which we have labeled Regulation of Antisociality was uniquely loaded by items reflecting regulation of antisocial traits. Loading on both factors (but generally a little more strongly on Sexual Self-Management) were D2 sexual compulsivity, D5 cognitive distortions, D7 emotional control, and D8 insight. One way to characterize this difference is that while the Treatment Responsivity factor assessed as an LTV prior to treatment correlates only weakly (.20–.30) with the Sexual Deviance and Criminality factors, change on Treatment Responsivity items co-occurs with either change in Sexual Deviance items or change in Criminality items to such a degree that the Treatment Responsivity factor merges into the other two factors.

That the structure of change in LTVs might be different from the structure of LTVs themselves is suggested by Thornton’s TDR since it represents change as resulting from the development of regulation processes. More specifically, how can the structure of treatment induced change be understood? The TDR proposes that schema regulation is likely to develop when individuals’ interactions with their environments provide feedback that a previously useful schema is now less functional. For someone living in a treatment regime while participating in treatment sessions there will be two sources of feedback. First, the broader regime will press the individual to comply with a sentence plan, follow rules, avoid conflicts with staff and so forth. As a consequence, behavioral expression of antisocial traits will generate corrective feedback from the regime. In contrast, sexual deviance can be largely invisible to the regime at large but will be a primary focus of a sexual offense-specific treatment group. According to the theory, how people respond to feedback will depend on the relationship they have with the sources of the feedback. The relationship may be antagonistic, in which case pressure from environmental feedback may be resisted, or it may be more collaborative, in which case feedback will be taken as a useful source of coaching regarding how to navigate the environment more effectively. Thus, differences between people at the end of treatment in the Regulation of Antisociality factor can be understood as reflecting the quality of the relationship the individual has with the regime in general while differences between people at the end of treatment in the Sexual Self-Management factor can be understood as reflecting the quality of relationships between individual and their therapist(s).

Can this general explanation also make sense of the items that load on both factors? It would make sense for an item to load on both factors if the behavioral expression of the item would generate corrective feedback both in a treatment group and in the treatment regime. This is plausible for each of the four items involved. For instance, considering the sexual compulsivity item, the person who responds to their world in a hypersexual way, using sexual behavior as a leading source of reward and as a way of coping with stress, may get corrective feedback inside a treatment group (especially if they disclose excessive sexual fantasizing or sexualized ways of interpreting events) but they will also get feedback from the institutional environment if their hypersexuality leads to flirting or sexual behavior with staff or other inmates, including acts such as “accidentally” exposing themselves to staff. Similarly, poor regulation of emotions is a natural focus in a treatment group, but it will also generate feedback from the regime if emotional dysregulation leads to acting out. Finally, while cognitive distortions and lack of insight are a primary focus in treatment group, these may also be displayed in the context of clashes with the regime.

Univariate analyses of the predictive value of individual dynamic items generally gave results supportive of their relevance to sexual recidivism. Univariate analyses of pretreatment dynamic ratings found statistically significant associations with sexual recidivism for all except three of the 17 dynamic items. Univariate analyses of posttreatment dynamic ratings found statistically significant associations with increased sexual recidivism for all items except D3 offense planning. Similar analyses for change ratings (with pretreatment ratings partialled out) found change to be significantly associated with reduced sexual recidivism for all items. Overall, these results are supportive of the item selection decisions that were made in constructing the VRS-SO and, remembering that a particular dynamic risk score may be achieved through different items, nearly all items being predictive is reassuring. It is interesting that change was substantively and significantly protective even for items like D5 cognitive distortions for which pretreatment ratings had little predictive value. An anonymous reviewer suggested that this apparently paradoxical result might actually be part of a more general pattern in which the predictiveness of pretreatment ratings would be less for the items that showed the greatest change during treatment. A supplementary analysis (Supplemental Table S3) supported this idea. This implies that targeting these items will still be beneficial. More generally, the significance of change for all the items reinforces the value of the VRS-SO as a tool for treatment planning.

Analyses of incremental prediction led to a complex pattern of results that presents further demands on theory. Focusing first on pretreatment dynamic items as these most cleanly reflect LTVs, items can be categorized as either factor-dominant or item-dominant. For factor-dominant items when sexual recidivism is jointly predicted by the item and by the sum of the other items from the parent factor to which the item belongs it is the parent factor that has most of the predictive value while some of the items even have negative predictive value. Thus, for these items, it is the variance they share with the parent factor that primarily contributes to risk. In contrast, for item-dominant items, the item retains its predictive value while the parent factor’s prediction shrinks once the item is controlled. This suggests that the factor works to predict through the variance it shares with these items. They are, as it were, funnels through which the effect of the factor operates.

Using this classification system to examine the Sexual Deviance factor, deviant sexual preference and offense planning emerge as factor-dominant. Notably, the Cox regression weight for D3 offense planning actually becomes significantly negative when its parent factor is controlled. We can understand this as planning offenses requiring some sustained motivation to offend but once this is partialled out, offending impulsively may be riskier since it will be less constrained by consequences. For D16 deviant sexual preference, we can more simply say that its predictive power results from variance it shares with the parent factor. In contrast D1 sexually deviant lifestyle and D12 sexual offense cycle emerge as item-dominant. Putting these interpretations together, D16 deviant sexual preferences and the other elements of the Sexual Deviance factor are only predictive when they pervasively permeate and organize the person’s life in the way captured by the sexually deviant lifestyle and sexual offense cycle items.

Turning to Treatment Responsivity items, D8 insight and D5 cognitive distortions emerge as factor-dominant; indeed, like D3 offense planning, cognitive distortions actually acquires a negative weight once its parent factor is controlled. In contrast, D11 release to high-risk situations and D15 treatment compliance are item-dominant. All four items on this factor involve some combination of cognitive insight and motivation to manage risk but they differ in the degree to which these two aspects are weighted with motivation being more central to release to high-risk situations and treatment compliance. The results suggest that it is the ability of these items to tap motivation to avoid future offending that gives this factor its predictive power. Lack of insight and cognitive distortions seem only to matter to the extent that they preclude motivation.

Classifying Criminality items in the same way, D4 criminal personality, D6 interpersonal aggression, D9 substance abuse, and D13 impulsivity emerge as factor-dominant. None of the items are truly item-dominant; the parent Criminality factor always remains substantively predictive regardless of which item is controlled. Thus, prediction from Criminality items primarily operates at the factor level. However, D14 compliance with community supervision and D10 community support emerge as having incremental value beyond the parent factor. This may reflect that these items relate to the individual’s engagement with potential external protective factors in the way envisaged in instruments like the SAPROF-SO (Willis et al., 2020) while the other criminality items and the parent factor itself is primarily a feature of the individual himself.

What do these results say about whether VRS-SO items or broader factors should be the focus of theoretical analysis? Neither possibility can be dismissed. Risk does not solely operate at the factor level, nor does it solely operate at the level of individual items. Theoretical analysis of dynamic risk will need to operate at both levels and, make sense of why and how some items operate primarily through factors while others operate as funnels through which broader factors have their influence.

### Summary of Implications for Theory Development

The primary intent of this article was to develop empirical findings that might guide future theory development. In light of our results, taken in conjunction with previous research, we suggest that future theoretical development should be consistent with and make sense of following:

1. There being at least three broad dimensions (Sexual Deviance, Criminality, and Treatment Responsivity) that run through pretreatment LTVs;
2. Similar factors occurring in static and pretreatment dynamic items;
3. Change not simply being movement along the same dimensions that run through pretreatment LTVs;
4. The interplay between item and factor levels.

Although our focus is on further development of the TDR, there are other promising theoretical frameworks and we invite proponents of the DRRF (Ward & Fortune, 2016), Life Course Theory (e.g., Lussier & McCuish, 2020), or the Motivation-Facilitation Model (Seto, 2019) to take up the challenge of developing theory in a way that explains all these findings.

### Summary of Implications for Future Research

Future research might seek to replicate the core findings reported here or to see whether they can be conceptually replicated if risk items are operationalized in other ways. Future research might seek to explore the two change factors. This might include identifying pretreatment indicators or contextual factors that would affect the form that change takes. Would the structure of change be the same for treatment that takes place in the community or for treatment that had a different focus? For example, would participation in the kind of prison Therapeutic Community described by Cullen et al. (1997) where the focus is almost entirely on processing life in the community yield a single dimension of change?

There are particular implications for those seeking to evaluate treatment programs. Short of random allocation research designs will be defective if they only consider static risk indicators when trying to remove unwanted differences between treatment and comparison groups. This applies equally to traditional regression methods and to the newer propensity score matching methods. Quite simply, controlling the Sexual Criminality factor does not control for the two factors found in sexual offense-specific dynamic items. Given the difficulty of rating dynamic items from retrospective file information for individuals who are not participating in treatment, new approaches will be required.

### Summary of Implications for Clinical Practice

The present results have implications for both the assessment of treatment needs and the focusing of clinical effort during treatment. The fact that change on each item was related to reduced recidivism gives more confidence in using items to identify specific treatment needs. Furthermore, where the predictive effect of a parent factor is mediated through particular items this suggests that during treatment related to a parent factor, particular attention should be paid to the targeting of the aspects of the factor tapped by these items. The results could also helpfully inform posttreatment assessment by characterizing change in terms of the two change factors identified here.

### Limitations

There are three primary sets of limitations from the present research. First, a potential limitation is that this article only explores change arising from a particular change agent, sexual offense-specific treatment. There are other potential change agents and the present results do not inform as to whether change associated with aging, improvement in community supports, or time in the community free of offending would have the same characteristics as treatment-induced change. The second limitation is that the present study featured a single risk tool, the VRS-SO, with data collected from two countries. Further research is needed to replicate and extend the present study findings regarding the TDR employing other tools of static and dynamic risk (e.g., STABLE-2007; SOTIPS), and in other samples and settings, to extend the reach and confidence in the range of applications of the TDR. Third, the present research does not afford empirical exploration of how the construct of agency is employed in the TDR. A different methodology would be needed for that to be addressed. One possibility might be a prospective longitudinal study that explored how components from the Reasoned Action Approach (Fishbein & Ajzen, 2010) varied as a function of changes in dynamic risk as measured by the VRS-SO.

## Supplemental Material

sj-pdf-1-sax-10.1177_10790632211002858 – Supplemental material for Understanding the Latent Structure of Dynamic Risk: Seeking Empirical Constraints on Theory Development Using the VRS-SO and the Theory of Dynamic Risk Click here for additional data file. Supplemental material, sj-pdf-1-sax-10.1177_10790632211002858 for Understanding the Latent Structure of Dynamic Risk: Seeking Empirical Constraints on Theory Development Using the VRS-SO and the Theory of Dynamic Risk by Mark E. Olver, David Thornton and Sarah M. Beggs Christofferson in Sexual Abuse: A Journal of Research and Treatment

## Notes

1 For the New Zealand subsample, only VRS-SO static total scores were inputted for analysis following data collection and thus individual static item scores were not available for examination.

2 The Cox regressions were repeated while also censoring violent recidivists who did not sexually reoffend as a conservative control for time at risk in the community, given that time incarcerated for new nonsexual offenses was not consistently available for each subsample to adjust time at risk. The substantive predictor-criterion findings did not change, and in some instances improved slightly. Given that most new prison sentences were of a determinate nature and did not entail the individual being incarcerated for the duration of the follow-up, the original survival time variable (adjusted for pretrial custody but not incarceration for nonsexual offenses) was retained as a more accurate, although imperfect, estimate of time at risk in the community.

3 We did not impose controls for offense history or age in the change-outcome individual predictor analyses. Olver et al. (2020) found that age at release did not moderate VRS-SO change associations with sexual or violent recidivism; the youngest cohort of men (aged <30 years) registered significantly less change than men aged 40–49 years, otherwise there were no significant differences in change among age cohorts. The results of fixed-effects meta-analysis across age cohorts showed that there was no significant variability in effect size magnitudes for change associations with recidivism outcome.
