# Supplementary material for: Understanding the Latent Structure of Dynamic Risk: Seeking Empirical Constraints on Theory Development Using the VRS-SO and the Theory of Dynamic Risk
Source: Sex Abuse. 2021 Apr 5;34(2):227–54. doi: 10.1177/10790632211002858 (PMC8848056; doi:10.1177/10790632211002858)
Supplement: sj-pdf-1-sax-10.1177_10790632211002858 – Supplemental material for Understanding the Latent Structure of Dynamic Risk: Seeking Empirical Constraints on Theory Development Using the VRS-SO and the Theory of Dynamic Risk [file sj-pdf-1-sax-10.1177_10790632211002858.pdf]

Supplemental Table S1

VRS-SO-TDR Factor Intercorrelations

Factor	Age	Sexual criminality	General criminality	Sexual deviance
Sexual criminality	.187***			
General criminality	.369***	.320***		
Sexual deviance	-.122***	.466***	.086**	
Treatment responsivity	.007	.152***	.307***	.239***

Note: ** $p < .01$, *** $p < .001$; Listwise $N = 928$

Supplemental Table S2

Incremental Predictive Validity for Sexual Recidivism by Pre and Posttreatment Rated Dynamic Items Controlling for Relevant Static and Dynamic Items

Regression model	Pretreatment						Posttreatment					
	B	*SE*	*p*	e^B	*95% CI*		*B*	*SE*	*p*	e^B	*95% CI*	
D1 Sexually deviant lifestyle	.20	.10	**.049**	1.21	1.001	1.48	.28	.12	**.016**	1.32	1.05	1.65
Sexual deviance w/o D1	-.05	.03	.136	0.95	0.90	1.02	-.03	.04	.365	0.97	0.90	1.04
Sexual criminality	.12	.02	**<.001**	1.13	1.08	1.17	.11	.02	**<.001**	1.11	1.07	1.16
D2 Sexual compulsivity	.03	.09	.699	1.03	0.87	1.22	.06	.10	.559	1.06	0.87	1.29
Sexual deviance w/o D2	.00	.03	.890	1.00	0.94	1.05	.03	.03	.427	1.03	0.96	1.09
Sexual criminality	.12	.02	**<.001**	1.13	1.08	1.17	.11	.02	**<.001**	1.11	1.07	1.16
D3 Offense planning	-.18	.08	**.028**	0.84	0.71	.93	-.17	.10	.079	0.85	0.70	1.02
Sexual deviance w/o D3	.05	.03	.070	1.05	1.00	1.11	.09	.03	**.010**	1.09	1.02	1.15
Sexual criminality	.11	.02	**<.001**	1.12	1.07	1.17	.10	.02	**<.001**	1.10	1.06	1.15
D12 Sexual offending cycle	.09	.09	.272	1.10	0.93	1.30	.19	.10	.067	1.20	0.99	1.47
Sexual deviance w/o D12	-.01	.03	.580	0.99	0.94	1.04	.00	.03	.095	1.00	0.95	1.06
Sexual criminality	.11	.02	**<.001**	1.12	1.07	1.17	.10	.02	**<.001**	1.11	1.06	1.15
D16 Deviant sexual preference	-.07	.09	.451	0.93	0.78	1.12	-.11	.10	.281	0.90	0.74	1.09
Sexual deviance w/o D16	.02	.03	.453	1.02	0.96	1.09	.07	.04	**.044**	1.08	1.002	1.15
Sexual criminality	.12	.02	**<.001**	1.13	1.08	1.17	.11	.02	**<.001**	1.12	1.07	1.16
D4 Criminal personality	.03	.08	.763	1.03	0.87	1.20	.04	.10	.652	1.04	0.87	1.26
Criminality w/o D4	.11	.03	**<.001**	1.11	1.05	1.18	.15	.03	**<.001**	1.16	1.09	1.23
S7 sentencing dates	.20	.08	**.019**	1.22	1.03	1.43	.15	.08	.060	1.16	0.99	1.36
D6 Interpersonal aggression	.12	.09	.188	1.13	0.94	1.35	.13	.10	.214	1.14	0.93	1.39
Criminality w/o D6	.09	.03	**.005**	1.09	1.03	1.16	.13	.03	**<.001**	1.14	1.07	1.22
S7 Sentencing dates	.20	.08	**.016**	1.22	1.04	1.44	.16	.08	**.047**	1.17	1.002	1.37
D9 Substance abuse	.02	.07	.770	1.02	0.89	1.16	.09	.08	.248	1.10	0.94	1.28
Criminality w/o D9	.11	.03	**<.001**	1.11	1.06	1.17	.14	.03	**<.001**	1.15	1.09	1.21
S7 Sentencing dates	.21	.08	**.013**	1.23	1.05	1.45	.17	.08	**.041**	1.18	1.01	1.38
D10 Community support	.13	.09	.172	1.13	0.95	1.36	.21	.10	**.030**	1.24	1.02	1.50
Criminality w/o D10	.09	.03	**<.001**	1.09	1.04	1.15	.12	.03	**<.001**	1.13	1.07	1.19
S7 Sentencing dates	.20	.08	**.015**	1.22	1.04	1.44	.16	.08	**.044**	1.18	1.004	1.38
D13 Impulsivity	-.08	.08	.379	0.93	0.78	1.10	-.02	.10	.839	0.98	0.80	1.20
Criminality w/o D13	.13	.03	**<.001**	1.14	1.08	1.21	.16	.03	**<.001**	1.18	1.11	1.25
S7 Sentencing dates	.18	.08	**.032**	1.20	1.02	1.41	.15	.08	.072	1.16	0.99	1.35
D14 Compliance with com. sup.	.40	.09	**<.001**	1.49	1.25	1.77	.46	.10	**<.001**	1.58	1.31	1.92
Criminality w/o D14	.03	.03	.263	1.03	0.98	1.09	.07	.03	**.031**	1.07	1.01	1.13
S7 Sentencing dates	.16	.08	.060	1.17	0.99	1.37	.11	.08	.168	1.12	0.95	1.31
D5 Cognitive distortions	-.18	.09	**.033**	.83	.70	.99	-.17	.10	.080	.84	.69	1.02
Txt Responsivity w/o D5	.24	.04	**<.001**	1.27	1.19	1.37	.27	.04	**<.001**	1.32	1.22	1.42
D8 Insight	.10	.10	.349	1.10	.90	1.35	.14	.11	.186	1.16	.93	1.43
Txt Responsivity w/o D8	.17	.04	**<.001**	1.19	1.10	1.28	.19	.04	**<.001**	1.21	1.11	1.31
D11 Released to HRS	.32	.08	**<.001**	1.38	1.19	1.60	.39	.09	**<.001**	1.48	1.25	1.75
Txt Responsivity w/o D11	.09	.04	**.020**	1.09	1.01	1.18	.09	.04	**.022**	1.10	1.01	1.18
D15 Treatment compliance	.41	.08	**<.001**	1.50	1.29	1.75	.44	.09	**<.001**	1.55	1.31	1.85
Txt Responsivity w/o D15	.05	.04	.219	1.05	.97	1.14	.08	.04	**.049**	1.08	1.00	1.17

Note: *N*s = 1,050 to 1,058

Supplemental Table S3

Dynamic Item Descriptive Statistics (Means) Rank Ordered by Pretreatment Hazard Ratio

Dynamic item		Pre *M*	Post *M*	Change *M*	Pre HR
D14	Compliance with community supervision	1.21	1.08	.12	1.65
D15	Treatment compliance	0.87	0.77	.11	1.58
D11	Released to high risk situations	1.46	1.31	.15	1.47
D6	Interpersonal aggression	1.49	1.29	.20	1.44
D10	Community support	1.52	1.37	.16	1.39
D8	Insight	2.03	1.60	.43	1.34
D4	Criminal personality	0.96	0.87	.09	1.32
D13	Impulsivity	1.27	1.12	.15	1.31
D7	Emotional control	1.94	1.62	.32	1.28
D1	Substance abuse	1.43	1.20	.23	1.26
D9	Sexually deviant lifestyle	1.48	1.28	.19	1.26
D12	Sexual offending cycle	1.80	1.47	.33	1.24
D2	Sexual compulsivity	1.25	1.10	.15	1.18
D16	Deviant sexual preference	1.43	1.26	.18	1.12
D17	Intimacy deficits	1.47	1.28	.19	1.11
D5	Cognitive distortions	2.00	1.64	.35	1.02
D3	Offense planning	1.59	1.30	.29	1.01

Pearson r = -.445, *p* = .074
Spearman's rho = -.464, *p* = .061

Supplemental Table S4

Cox Regression Survival Analyses: Incremental Predictive Validity for VRS-SO Dynamic and Change Factor Scores (N = 1,288)

Variable	*B*	*SE*	*p*	e^B	*95% CI*
Sexual deviance (pre)	.075	.021	<.001	1.078	1.035, 1.123
Sexual deviance (change)	-.263	.097	.007	0.769	0.636, 0.929
Criminality (pre)	.156	.023	<.001	1.169	1.118, 1.222
Criminality (change)	-.362	.097	<.001	0.696	0.576, 0.841
Treatment responsivity (pre)	-.025	.035	.474	0.975	0.910, 1.045
Treatment responsivity (change)	.156	.113	.167	1.169	0.937, 1.458
